# Species extinction thresholds in the face of spatially correlated periodic disturbance

**DOI:** 10.1038/srep15455

**Published:** 2015-10-20

**Authors:** Jinbao Liao, Zhixia Ying, David E. Hiebeler, Yeqiao Wang, Takenori Takada, Ivan Nijs

**Affiliations:** 1 Ministry of Education’s Key Laboratory of Poyang Lake Wetland and Watershed Research, Jiangxi Normal University, Ziyang Road 99, 330022 Nanchang, China; 2 State Key Laboratory of Vegetation and Environmental Change, Institute of Botany, Chinese Academy of Sciences, Beijing 100093, China; 3 Department of Mathematics and Statistics, University of Maine, 333 Neville Hall, Orono, ME 04469, USA; 4 Laboratory of Mathematical Ecology, Graduate School of Earth Environmental Science, Hokkaido University, 060-0810 Sapporo, Japan; 5 Research Group Plant and Vegetation Ecology, Department of Biology, University of Antwerp (Campus Drie Eiken), Universiteitsplein 1, B-2610 Wilrijk, Belgium

## Abstract

The spatial correlation of disturbance is gaining attention in landscape ecology, but knowledge is still lacking on how species traits determine extinction thresholds under spatially correlated disturbance regimes. Here we develop a pair approximation model to explore species extinction risk in a lattice-structured landscape subject to aggregated periodic disturbance. Increasing disturbance extent and frequency accelerated population extinction irrespective of whether dispersal was local or global. Spatial correlation of disturbance likewise increased species extinction risk, but only for local dispersers. This indicates that models based on randomly simulated disturbances (e.g., mean-field or non-spatial models) may underestimate real extinction rates. Compared to local dispersal, species with global dispersal tolerated more severe disturbance, suggesting that the spatial correlation of disturbance favors long-range dispersal from an evolutionary perspective. Following disturbance, intraspecific competition greatly enhanced the extinction risk of distance-limited dispersers, while it surprisingly did not influence the extinction thresholds of global dispersers, apart from decreasing population density to some degree. As species respond differently to disturbance regimes with different spatiotemporal properties, different regimes may accommodate different species.

Ecologists have long explored the effects of disturbance on the abundance and persistence of species populations^1^,^2^,^3^,^4^,^5^,^6^. One of the fundamental characteristics of disturbance is its discrete nature in time and space^4^. Disturbance events can be biotic (e.g., pest outbreaks and grazing) or abiotic (e.g., flooding, fires and drought), but also anthropogenic such as selective harvesting, trampling and man-induced climate extremes^7^. To date, an increasing body of research, including both theoretical and empirical, has focused on understanding how different aspects of disturbance influence population dynamics as well as species coexistence^6^,^8^,^9^,^10^,^11^,^12^,^13^. In these studies, the individual physical characteristics of a disturbance regime are often characterized by frequency and intensity. Much research thus far has ignored the spatial correlation of disturbance by assuming that all individuals in a landscape are disturbed. However, many disturbances (e.g., fire, drought, flooding, etc.) occur spatially aggregated.

Recent theoretical work established that spatially correlated disturbance may act negatively on population dynamics^14^,^15^,^16^,^17^,^18^,^19^. Despite these studies, it is not well understood how spatially correlated disturbance interacts with species traits in modifying species extinction thresholds. Probably, species with different traits (e.g., dispersal characteristics or intraspecific competitive ability) respond differently to the same type of disturbance, and therefore exhibit distinct extinction responses. Furthermore, the critical biotic or abiotic trait parameter values above/below which a population goes extinct under spatially correlated disturbance have not been characterized.

In this study, we explore these interactive effects of spatially correlated periodic disturbance and species traits on species extinction thresholds with a pair approximation model. Pair approximation models have proved useful in describing spatial neighbouring correlation with a variety of lattice models^16^,^19^,^20^,^21^,^22^,^23^,^24^. In the model, a disturbance is defined as any discrete event that removes individuals and forms gaps which can be recolonized by individuals of the same or different species^25^,^26^. We consider three independent aspects of disturbance, i.e., its periodicity (frequency), disturbed area (extent) and spatial pattern (spatial correlation). For simplicity, we assume that disturbance events occur within a single time step (i.e., pulse disturbance) and only cause mortality of disturbed individuals, while ignoring effects on species recruitment and habitat quality. Different from previous modelling work^14^,^15^,^16^,^17^,^18^,^19^, we mainly focus on seeking species extinction thresholds in the face of spatially correlated disturbance, as it can provide critical reference for species conservation. In particular, extinction thresholds can serve as early warning signals for future species extinctions, provide a tool to conservation ecologists towards identifying the most sensitive species, and guide priority setting in conservation efforts.

## Results

**Figure 1:**
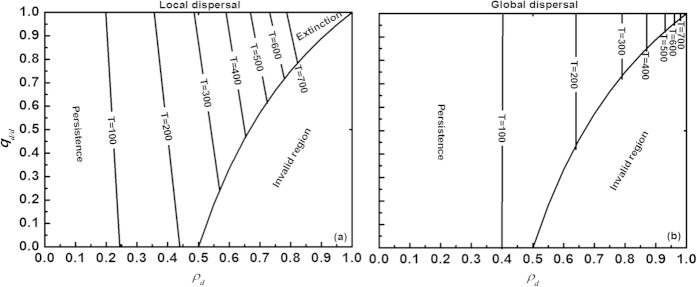
Interactive effects of spatially correlated periodic disturbance and dispersal traits on species extinction thresholds. Three principal aspects of disturbance are varied: disturbance periodicity (T = 100, 200, 300···700), disturbance extent (0 < *ρ*_*d*_ < 1), and spatial clumping of the disturbed area (*q*_*d/d*_ ∈ 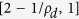). Two types of dispersal are simulated: local (**a**) and global (**b**). The boundary line dividing the region of species persistence (left region) and extinction (right region) varies with disturbance periodicity (T). As the range of spatial correlation of disturbance (*q*_*d/d*_) shrinks with increasing disturbance extent (*ρ*_*d*_), each panel also has an invalid region according to equation (3). Parameter values: intrinsic birth rate *α* = *β* = 0.01, intrinsic mortality rate *m* = 0.005 and species sensitivity to crowding (i.e., intraspecific competition coefficient) *γ* = 0.

**Figure 2:**
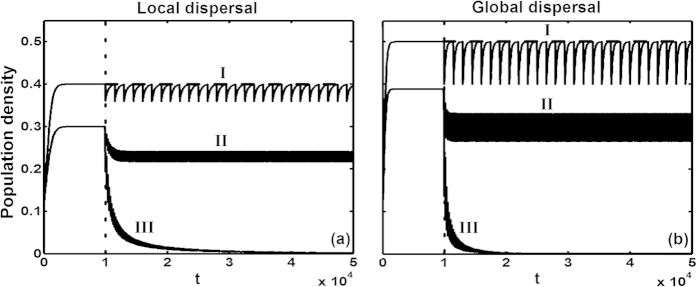
Three typical types of dynamic population behavior (see description in Methods) following spatially correlated periodic disturbance after *t* > 10000 (dotted line), regardless of dispersal traits. Before disturbance (0 ≤ *t* ≤ 10000), the simulation was run until populations reached equilibrium density. Panel (**a**) with local dispersal: (I) species parameters (*α*, *m*, *γ*) = (0.01, 0.005, 0) and disturbance parameters (*ρ*_*d*_, *q*_*d/d*_, T) = (0.1, 0.1, 1500); (II) (*α*, *m*, *γ*) = (0.01, 0.005, 0.002) and (*ρ*_*d*_, *q*_*d/d*_, T) = (0.1, 0.1, 200); (III) (*α*, *m*, *γ*) = (0.01, 0.005, 0.002) and (*ρ*_*d*_, *q*_*d/d*_, T) = (0.35, 0.35, 200). Panel (b) with global dispersal: (I) (*β*, *m*, *γ*) = (0.01, 0.005, 0) and (*ρ*_*d*_, *q*_*d/d*_, T) = (0.2, 0.2, 1500); (II) (*β*, *m*, *γ*) = (0.01, 0.005, 0.003) and (*ρ*_*d*_, *q*_*d/d*_, T) = (0.2, 0.2, 200); (III) (*β*, *m*, *γ*) = (0.01, 0.005, 0.003) and (*ρ*_*d*_, *q*_*d/d*_, T) = (0.65, 0.65, 200).

We first investigated the interactive effects of different aspects of the disturbance regime on species extinction, while taking account of species dispersal traits (Fig. 1). Both under local and global dispersal, enlarging disturbance periodicity (T) increased the region of species survival, but with less increment at longer periodicity (see Fig. 2). However, species dispersal substantially modified these effects of spatially correlated disturbance on species extinction risk. Under local dispersal, increasing the disturbance extent (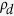) and the spatial correlation (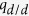) both promoted the species extinction risk. For instance, increasing 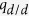 at a fixed T required more undisturbed area (1 − 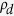) for the species to persist. Similar to local dispersal, species under global dispersal more easily went extinct (i.e., more closely approached the extinction threshold curves) as the disturbed area increased. Yet, global dispersers were not influenced by the spatial clumping of the disturbed area, because of their random establishment. Due to their dispersal superiority, species with global dispersal tolerated more severe disturbance relative to local dispersers (compare Fig. 1a,b).

**Figure 3:**
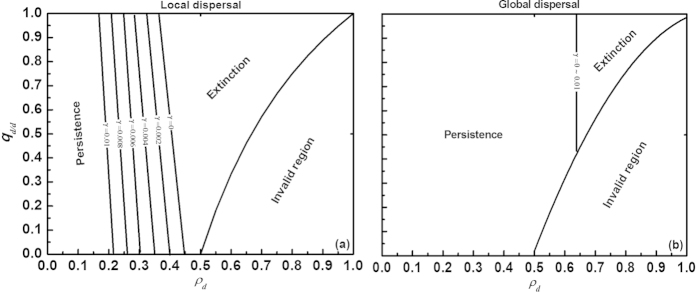
Interactive effects of spatially correlated disturbance and intraspecific competition (*γ = *0, 0.002, 0.004···0.01) on species extinction thresholds at fixed periodicity T = 200, considering both local (a) and global (b) dispersal. Similar to Fig. 1, two principal aspects of disturbance are varied: disturbance extent *ρ*_*d*_ ∈ [0, 1] and spatial correlation *q*_*d/d*_ ∈ 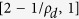. The boundary line separating species persistence (left region) from extinction (right region) shifts with the intraspecific competition coefficient (*γ*). Invalid region: see equation (3). Other parameters: see Fig. 1.

Next we explored whether and how intraspecific competition modifies the species extinction thresholds at fixed disturbance periodicity (T = 200) (Fig. 3). Again, the spatial aggregation of disturbance promoted the extinction risk of local dispersers, while it had no influence on the extinction thresholds of global dispersers. Interestingly, increasing intraspecific competition (*γ*) reduced the survival area of local dispersers, while the extinction risk of global dispersers was not changed. Most likely, the influence of intraspecific competition on global dispersers gradually disappeared with declining population size, while for local dispersers it was maintained because intraspecific clustering does not approach zero in a locally dispersing population even when the population density approaches zero.

**Figure 4:**
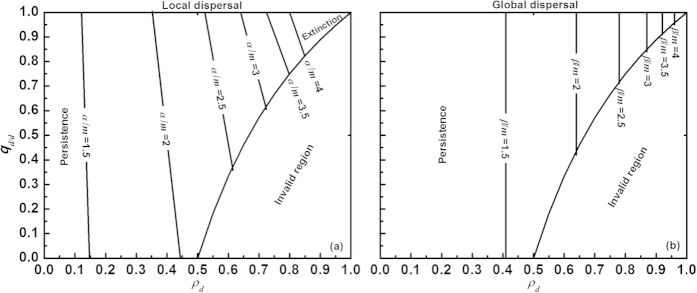
Interactive impacts of spatially correlated disturbance and species relative birth rate (*α/m* under local dispersal in panel (a) or *β/m* under global dispersal in panel (b)) on species extinction risk at fixed periodicity T = 200, again varying both disturbance extent (*ρ*_*d*_) and spatial correlation (*q*_*d/d*_). Intrinsic mortality was set to *m* = 0.005. The line between species persistence (left region) and extinction (right region) varies with relative birth rate (*α/m* or *β/m*). Invalid region: see equation (3). Parameter values: *α/m* = *β/m* = {1.5, 2, 2.5···4} and intraspecific competition coefficient *γ* = 0.

Finally, we tested the combined effects of species relative birth rate (*α/m* or *β/m*) and spatially correlated disturbance on the extinction risk of both local and global dispersers at fixed periodicity (T = 200), as shown in Fig. 4. Increasing relative birth rate enhanced species survival, but the effect levelled off at higher values. Species dispersal modified the effects of relative birth rate. Under local dispersal, the spatial correlation of disturbance promoted species extinction risk (i.e., the species tolerated less disturbed habitat), but more so at higher *α/m* (i.e., the slope of the extinction curves increased) (Fig. 4a). Under global dispersal, the spatial aggregation of disturbance did not alter extinction regardless of *β/m* (Fig. 4b). Relative to local dispersers, global dispersers tolerated more disturbed habitat area at *α/m* = *β/m* (compare Fig. 4a,b).

## Discussion

We developed a pair approximation model to explore species extinction risk in a lattice-structured landscape subject to spatially correlated periodic disturbance. Compared to non-spatial models^8^,^9^,^10^,^11^, the modelling novelty is that conditional probabilities were used to describe the spatial structure of disturbance, as well as neighbouring dispersal and intraspecific competition. Relative to using spatially explicit individual-based models (IBMs)^18^,^27^, using PA models to examine the spatial effect of disturbance on species survival is more efficient because they require less computation time; moreover, IBM results tend to be approximate with large deviations. Although we ignored spatial correlation beyond nearest neighbours (e.g., triplet local correlation^20^,^21^), several outcomes were similar to those of other spatial models (see further). By incorporating the essential spatial aspects, the PA approach can thus bridge the gap between non-spatial models and spatially explicit IBMs.

As intuitively expected, enhancing the extent and frequency (1/T) of disturbance accelerated species extinction irrespective of whether dispersal was local or global (Figs 1, 3 and 4), as confirmed by previous models^14^,^15^,^16^,^17^,^18^,^19^. When the disturbed area was more spatially correlated, the extinction of local dispersers became more likely, whereas the extinction thresholds for global dispersers were not influenced. Yet, the effects of disturbance extent generally outweighed those of spatial correlation, thereby playing a critical negative role in regulating species persistence. On the other hand, the dispersal traits strongly modified the impact of the disturbance extent on the species extinction thresholds. We found that the maximum allowable disturbed area below which global dispersers can persist is much larger than the corresponding maximum disturbed area for local dispersers (Figs 1, 3 and 4), suggesting that long-range dispersers can tolerate much more severe disturbance because of their dispersal superiority. From an evolutionary perspective, if two types of dispersal – local and global – evolve from a single population, then individuals performing local dispersal are more likely to become extinct facing strong spatially correlated disturbance than long-range dispersing individuals which can more easily adapt to the disturbance. As a result, spatially correlated disturbance could affect the evolution of species dispersal, favoring the long-range type (as confirmed by Kallimanis *et al*.^18^,^27^).

Why did spatial correlation of disturbance promote the extinction risk in our model^14^,^15^,^16^,^27^, but only for local dispersers (compare Fig. 1a,b)? Logically, spatially correlated disturbance tends to locally destroy a population, forming large openings that require more time to reach and recolonize especially for species with distance-limited dispersal, thus reducing the density of a locally disturbed population (cf. decreasing population growth rates^28^,^29^). Global dispersal with random establishment, on the other hand, can allow a population to rapidly exploit the creation of large gaps, thereby eliminating the negative influence of spatial correlation in disturbance (Fig. 1b). The fact that the species extinction risk under local dispersal was higher in our model when disturbance was more spatially aggregated (with 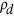 < 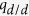) than randomly structured (with 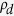 = 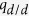; see Figs 1, 3 and 4), implies that models based on randomly synthesized disturbances (e.g., mean-field models and non-spatial models^8^,^9^,^10^,^11^) might underestimate real species extinction rates, as both natural and anthropogenic disturbances are rarely a completely random process. Different from spatial correlation of disturbance, an increasing spatial aggregation of fragmented habitat interestingly has an opposite (positive) effect on the persistence of local dispersers, irrespective of whether these fragmented landscapes are dynamic or static^15^,^23^,^30^,^31^. As explained in Liao *et al*.^23^, habitat connectivity provides opportunity for local recolonization, whereas habitat fragmentation promotes the risk of individuals locally dispersing from suitable to unsuitable sites.

Following periodic disturbance, intraspecific competition greatly enhanced the extinction rate of distance-limited dispersers, while it surprisingly had no influence on the extinction threshold of global dispersers (Fig. 3), apart from somewhat decreasing population density (see Fig. 2b where increasing species sensitivity to local crowding *γ* resulted in a lower equilibrium density). Logically, species that produce offspring around themselves (e.g., with vegetative, short-range clonal dispersal) generate conspecific clumping (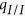 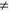 0) and therefore promote kin competition (*γ∙*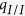 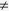 0) even at very low population density, thereby largely deceasing species persistence. This mechanism does not apply to global dispersal (e.g., long-range, wind-based dispersal of seeds). In particular, if a population with global dispersal that is suffering from severe disturbance approaches extinction (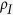 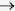 0), and intraspecific clumping tends to zero as well (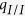 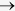 0) because of random establishment, then the intraspecific competition effect will disappear owing to the extremely low population levels (*γ∙*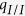 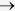 0). Consequently, long-range dispersers can tolerate more intense kin competition compared to distance-limited dispersers in the face of spatially correlated disturbance. Interestingly, this result is similar to Liao *et al*.^23^,^31^ in which species extinction thresholds were explored in static fragmented landscapes, further implying that kin competition can promote the extinction risk of local dispersers irrespective of whether landscapes suffer from dynamic or static disturbance.

In this model, we only considered two extreme types of dispersal for model simplicity and mathematical tractability, with local dispersal to four nearest neighbouring sites and global dispersal being random across the entire landscape. Both simplifying forms of dispersal are relatively restrictive, as most species actually follow a specific dispersal kernel (for example, Gaussian). However, both dispersal modes have long been used in ecological models and are considered meaningful to analyze dispersal effects on eco-dynamics^32^,^33^,^34^,^35^,^36^,^37^, as they can avoid excessively complex simulations and generate approximately the same results as more realistic dispersal modes. Therefore, our modelling results should be considered as representative for two extreme dispersal modes that delimit the continuum in between. Concerning ecological implications, our simulations are most relevant for pulse disturbances that occur nearly instantaneously—at least relative to the lifespan of plant individuals—such as extreme events (e.g., pest outbreaks, fire, flooding, drought, storms and heatwaves). Application of our model to disturbances that gradually deteriorate the environment and alter habitat quality should be executed with caution, for example, prolonged release of diluted pollutants which kill plant individuals slowly and even reduce habitat suitability for plant growth, is not mimicked. Another assumption in our simulations was periodic disturbance, hence stochastic disturbance that occurs irregularly was not considered. Our model framework can be extended though to such cases by using occurrence rate (1/T), so that disturbance occurs every T time steps on average, following a Poisson process.

Further study could also focus on model validation. Moloney and Levin^38^ already explored the effects of spatiotemporal autocorrelation in disturbance on plant population dynamics, in an annual, serpentine grassland. Extending this type of experiment to test the influence of spatially correlated disturbance (by manipulating disturbance extent, periodicity and spatial correlation) on the extinction risk of plant species with mainly clonal growth or seed dispersal, would be fairly straightforward to achieve in synthesized grassland mesocosms, for example, via artificial removal of plant individuals within the disturbed area. Understanding how disturbance regimes affect species persistence is important for ecological conservation, as different species (with different dispersal strategies, intraspecific competition or relative birth rate) exhibit diverse responses, and different aspects of disturbance (including extent, spatial correlation and periodicity) bring different consequences for extinction risk. Our results suggest that species extinction risk will enhance in future decades, when both the frequency and extent of extreme climatic events are likely to increase further under climate change (see IPCC^39^,^40^). Mitigating climate change is needed but may be too slow to prevent major losses, so more pathways for species adaptation have to be explored and developed. Possibly, reducing spatial correlation in disturbances may be a strategy from which biodiversity conservation can benefit (e.g., through human control of fire spreading, inundation, pest outbreaks, etc.). Therefore, analyzing spatiotemporal variation in disturbance may provide new insights into species conservation.

## Methods

### Pair approximation (PA) model

We model population dynamics in a lattice-structured landscape consisting of only one type of habitat. Each site can be either occupied by an individual (denoted by *I*) or empty (*E*). For model simplicity, two extreme dispersal traits – local and global – are considered, with local dispersal restricted to nearest neighbouring sites (e.g., clonal growth) and global dispersal being random across the whole landscape (e.g., long-range seed dispersal). Similar to Liao *et al*.^23^, the population dynamics of global density 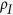 (without disturbance) follow


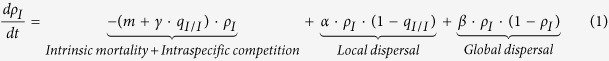


The first term on the right represents the sum of the intrinsic mortality (*m*) and the increased mortality (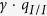) caused by intraspecific competition (for space and resources). The increased mortality is controlled by two factors: local crowding 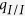 and species sensitivity (*γ*) to local crowding, where 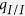 ∈ [0, 1] (the so-called local density) is the conditional probability of a neighbouring site of an individual *I* also being occupied by an individual *I*. In this model, we use the von Neumann neighbourhood consisting of the four orthogonal neighbours. According to previous studies^20^,^21^,^22^,^23^,^31^,^41^, the parameter 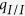 expresses the mean population crowding in the landscape with 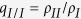. Here, the doublet density 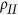 denotes the probability when randomly choosing a pair of nearest neighbours that both of them are *I*-sites. The second term denotes the population growth from local dispersal: *α* is the intrinsic birth rate via clonal growth, and (1 − 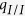) = 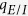 represents the average probability of empty *E*-sites surrounding the occupied sites in the entire landscape, as only two possible states – occupied (*I*) or empty (*E*) – exist for a randomly chosen neighbouring site of an occupied site. The last term represents the population growth from global dispersal: *β* is the intrinsic birth rate from random seed dispersal across the whole landscape, only considering seeds landing on empty sites while ignoring those landing on already occupied sites (see more details in Liao *et al*.^23^), and 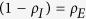 is the global density of empty sites.

According to Liao *et al*.^23^ and Ying *et al*.^24^, we derive the transition rate of 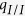 (see details in Supplementary A) as


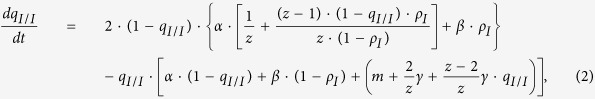


in which *z* = 4 with four nearest neighbours for each site, following von Neumann neighbourship.

Therefore, equations (1) and (2) with two variables (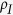 and 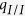) construct a closed mathematical system for spatially structured population dynamics in a homogeneous landscape, without disturbance.

### Spatially correlated periodic disturbance

**Figure 5:**
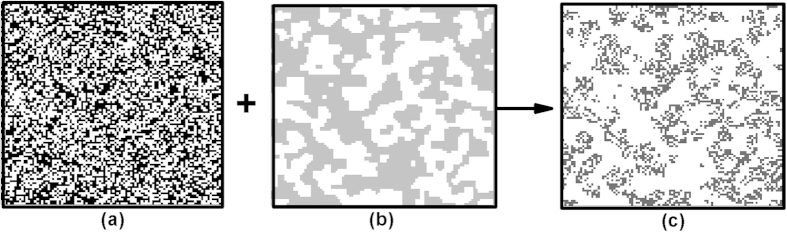
Illustration of population pattern formation in the face of spatially correlated disturbance in a lattice-structured landscape (black – individuals, white – empty sites, gray – disturbed area): (a) population pattern before disturbance (population density *ρ*_*I*_ = 0.5 and intraspecific clumping *q*_*I/I*_ = 0.5, with *ρ*_*I*_ = *q*_*I/I*_ representing random population distribution); (b) spatially correlated disturbance (disturbance extent *ρ*_*d*_ = 0.5 and its spatial correlation *q*_*d/d*_ = 0.9); (c) population pattern after disturbance.

We assume that disturbance events occur instantaneously, and that all individuals within the area of disturbed cells are killed in a single time step (see illustration in Fig. 5). In other words, when an occupied site is disturbed, it becomes empty but remains suitable for recolonization. Disturbance is applied in a spatially correlated way as a stochastic dynamic process, meaning that the location of a disturbance event is random, but with some degree of spatial aggregation. After the event, a new distribution of occupied sites is created.

The disturbance regime consists of three parameters: disturbance extent (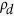 – the fraction of disturbed area in the entire landscape, *d* – a disturbed site), disturbance periodicity/interval (T), and spatial correlation (i.e., aggregation) of disturbance (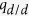). Similar to local crowding defined above, 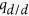 is the spatial clumping degree of disturbed sites with 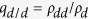, where 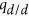 ∈ [0, 1] is the conditional probability of a neighbouring site of a disturbed site (*d*) also being disturbed, and 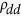 is the density of pair *d-d* sites, i.e., the probability when choosing a pair of nearest neighbours at random that both of them are disturbed. According to the algorithm of Hiebeler^30^, the range of 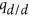 should follow


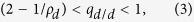


if only two possible site-states – disturbed (*d*) and undisturbed (*u*) – exist in a lattice-structured landscape. This means that the allowable clumping degree of disturbed sites depends on their global density. Following Hiebeler^30^, randomly structured disturbance has 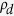 = 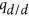, while the cases of 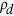 < 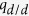 and 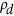 > 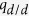 respectively represent spatially aggregated (correlated) disturbance and spatially over-dispersed disturbance.

After an instantaneous disturbance at 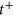, the transient population density is


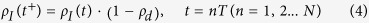


where 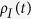 is population density at *t* before pulse disturbance. Given that the pulse disturbance does not influence habitat quality, the disturbed sites thus remain suitable for subsequent species recolonization. For example, if a pulse disturbance occurs and shapes the openings at *t*, then plant individuals can start recolonizing these openings in the next time step *t* + 1.

For the transient local density 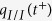 at 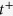, we have


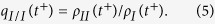


At 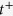, the transient pair density of *I-I* sites (i.e., the probability of the pair *I-I* sites keeping the same states after disturbance) should be


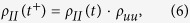


in which 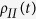 is the pair density of *I-I* sites at *t* before disturbance, and 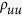 (*u* – an undisturbed site) is the probability that a pair of randomly chosen neighbouring sites are undisturbed. As defined above, we have 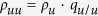, in which 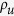 is the fraction of undisturbed sites, and 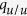 is the conditional probability that the neighbouring site of an undisturbed site is also undisturbed.

Following a disturbance event, a randomly chosen site in a lattice-structured landscape has two possible states – disturbed (*d*) and undisturbed (*u*). As the neighbouring sites can be either disturbed or undisturbed, we have the following constraints:


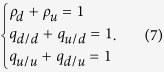


As a result, we obtain.


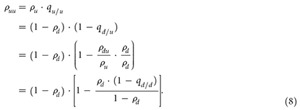


Combining equations (4~8) yields


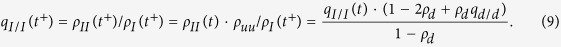


Therefore, equations (4) and (9) generally characterize the transient population pattern (global and local density) at time *t*^+^ after pulse disturbance.

### Numerical simulation cases

In this study, we answer three questions: (1) How do different aspects of the disturbance regime (disturbance extent 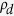, spatial correlation 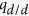 and periodicity T) influence species extinction thresholds, considering both local and global dispersal? These simulations are performed without intraspecific competition (*γ* = 0). (2) How do spatially correlated disturbance (varying 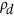 and 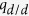) and intraspecific competition (*γ*) change species extinction risk, likewise including both local and global dispersal? Here the disturbance periodicity is set at T = 200, which shapes the range of moderate species survival. (3) Does variation in species relative birth rate *α/m* or *β/m* at fixed mortality (*m*) alter species extinction thresholds following disturbance? In this simulation, we again vary both 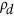 and 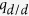 but keep T = 200, while ignoring intraspecific competition (*γ* = 0).

For all cases mentioned above, we first run simulations without disturbance until populations reach equilibrium density. Subsequent application of periodic disturbance yields three typical dynamic behaviors regardless of dispersal traits (Fig. 2): (I) the species returns to equilibrium density in each disturbance interval, resulting in periodic population fluctuation; (II) the species persists at a new steady state (periodic fluctuation) with a lower population density; (III) the species goes extinct (using 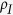 < 0.0001 as extinction threshold).

## Additional Information

**How to cite this article**: Liao, J. *et al.* Species extinction thresholds in the face of spatially correlated periodic disturbance. *Sci. Rep.* **5**, 15455; doi: 10.1038/srep15455 (2015).

## Supplementary Material

Supplementary Information

## References

[b1] Grime J. P. Evidence for the existence of three primary strategies in plants and its relevance to ecological and evolutionary theory. Am. Nat. 111, 1169–1194 (1977).

[b2] Connell J. H. Diversity in tropical rain forests and coral reefs. Science 199, 1302–1310 (1978). 17840770 10.1126/science.199.4335.1302

[b3] Huston M. A general hypothesis of species diversity. Am. Nat. 113, 81–101 (1979).

[b4] White P. S. & Pickett S. T. Natural disturbance and patch dynamics: an introduction. The ecology of natural disturbance and patch dynamics (Academic Press, Orlando, 1985).

[b5] Hastings A. & Wolin C. L. Within-patch dynamics in a metapopulation. Ecology 70, 1261–1266 (1989).

[b6] Colasanti R. L. & Grime J. P. Resource dynamics and vegetation processes: a deterministic model using two-dimensional cellular automata. Funct. Ecol. 7, 169–176 (1993).

[b7] Dornelas M. Disturbance and change in biodiversity. Phil. Trans. R. Soc. B 365, 3719–3727 (2010). 20980319 10.1098/rstb.2010.0295 PMC2982012

[b8] Chesson P. & Huntly N. The roles of harsh and fluctuating conditions in the dynamics of ecological communities. Am. Nat. 150, 519–553 (1997). 18811299 10.1086/286080

[b9] Mackey R. L. & Currie D. J. The diversity-disturbance relationship: is it generally strong and peaked? Ecology 82, 3479–3492 (2001).

[b10] Roxburgh S. H., Shea K. & Wilson J. B. The intermediate disturbance hypothesis: patch dynamics and mechanisms of species coexistence. Ecology 85, 359–371 (2004).

[b11] Fraterrigo J. M. & Rusak J. A. Disturbance-driven changes in the variability of ecological patterns and processes. Ecol. Lett. 11, 756–770 (2008). 18422637 10.1111/j.1461-0248.2008.01191.x

[b12] Miller A. D. & Chesson P. Coexistence in disturbance-prone communities: How a resistance-resilience trade-off generates coexistence via the storage effect. Am. Nat. 173, E30–E43 (2009). 10.1086/595750 29553822

[b13] Miller A. D., Roxburgh S. H. & Shea K. How frequency and intensity shape diversity–disturbance relationships. Proc. Natl. Acad. Sci. USA 108, 5643–5648 (2011). 21422284 10.1073/pnas.1018594108 PMC3078405

[b14] McCarthy M. A. & Lindenmayer D. B. Spatially-correlated extinction in a metapopulation model of Leadbeater’s Possum. Biodivers. Conserv. 9, 47–63 (2000).

[b15] Johst K. & Drechsler M. Are spatially correlated or uncorrelated disturbance regimes better for the survival of species? Oikos 103, 449–456 (2003).

[b16] Hiebeler D. E. Spatially correlated disturbances in a locally dispersing population model. J. Theor. Biol. 232, 143–149 (2005). 15498601 10.1016/j.jtbi.2004.08.007

[b17] Johst K., Gutt J., Wissel C. & Grimm V. Diversity and disturbances in the Antarctic megabenthos: feasible versus theoretical disturbance ranges. Ecosystems 9, 1145–1155 (2006).

[b18] Kallimanis A. S., Kunin W. E., Halley J. M. & Sgardelis S. P. Patchy disturbance favours longer dispersal distance. Evol. Ecol. Res. 8, 529–541 (2006).

[b19] Hiebeler D. E. & Morin B. R. The effect of static and dynamic spatially structured disturbances on a locally dispersing population. J. Theor. Biol. 246, 136–144 (2007). 17270218 10.1016/j.jtbi.2006.12.024

[b20] Matsuda H., Ogita N., Sasaki A. & Sato K. Statistical mechanics of population. Prog. Theor. Phys. 88, 1035–1049 (1992).

[b21] Harada Y. & Iwasa Y. Lattice population dynamics for plants with dispersing seeds and vegetative propagation. Res. Popul. Ecol. 36, 237–249 (1994).

[b22] Boots M. & Sasaki A. The evolutionary dynamics of local infection and global reproduction in host-parasite interactions. Ecol. Lett. 3, 181–185 (2000).

[b23] Liao J. *et al.* Species persistence in landscapes with spatial variation in habitat quality: A pair approximation model. J. Theor. Biol. 335, 22–30 (2013a). 23792314 10.1016/j.jtbi.2013.06.015

[b24] Ying Z. *et al.* Species coexistence in a lattice-structured habitat: Effects of species dispersal and interactions. J. Theor. Biol. 359, 184–191 (2014). 24937800 10.1016/j.jtbi.2014.05.048

[b25] Sousa W. P. The role of disturbance in natural communities. Annu. Rev. Ecol. Syst. 15, 353–391 (1984).

[b26] Townsend C. R. & Hildrew A. G. Species traits in relation to a habitat templet for river systems. Freshwater Biol. 31, 265–275 (1994).

[b27] Kallimanis A. S., Kunin W. E., Halley J. M. & Sgardelis S. P. Metapopulation extinction risk under spatially autocorrelated disturbance. Conserv. Biol. 19, 534–546 (2005).

[b28] Case T. J. An illustrated guide to theoretical ecology. (Oxford University Press, 2000).

[b29] Miller A. D., Reilly D., Bauman S. & Shea K. Interactions between frequency and size of disturbance affect competitive outcomes. Ecol. Res. 27, 783–791 (2012).

[b30] Hiebeler D. E. Populations on fragmented landscapes with spatially structured heterogeneities: landscape generation and local dispersal. Ecology 81, 1629–1641 (2000). 10.1007/s00285-006-0054-6 17151885

[b31] Liao J. *et al.* Modelling plant population size and extinction thresholds from habitat loss and habitat fragmentation: Effects of neighbouring competition and dispersal strategy. Ecol. Model. 268, 9–17 (2013b).

[b32] Hastings A. & Harrison S. Metapopulation dynamics and genetics. Annu. Rev. Ecol. Syst. 25, 167–188 (1994).

[b33] Hastings A. & Gavrilets S. Global dispersal reduces local diversity. Proc. R. Soc. B 266, 2067–2070 (1999). 10.1098/rspb.1999.0888 PMC1690330 10902543

[b34] Tilman D., Lehman C. & Kareiva P. Population dynamics in spatial habitats. Spatial Ecology: the role of space in population dynamics and interspecific interactions (Princeton University Press, 1997).

[b35] Johst K., Brand R. & Eber S. Metapopulation persistence in dynamic landscapes: the role of dispersal distance. Oikos 98, 263–270 (2002).

[b36] Bertuzzo E. *et al.* Spatial effects on species persistence and implications for biodiversity. Proc. Natl. Acad. Sci. USA 108, 4346–4351 (2011). 21368181 10.1073/pnas.1017274108 PMC3060266

[b37] Fennell M., Murphy J. E., Armstrong C., Gallagher T., Osborne B. Plant spread simulator: A model for simulating large-scale directed dispersal processes across heterogeneous environments. Ecol. Model. 230, 1–10 (2012).

[b38] Moloney K. A. & Levin S. A. The effects of disturbance architecture on landscape-level population dynamics. Ecology 77, 375–394 (1996).

[b39] IPCC. Climate change 2014: Impacts, adaptation, and vulnerability (Cambridge University Press, 2014a).

[b40] IPCC. Climate change 2014: Mitigation of climate change (Cambridge University Press, 2014b).

[b41] Lloyd M. Mean crowding. J. Anim. Ecol. 36, 1–30 (1967).

