# Supplementary material for: Species extinction thresholds in the face of spatially correlated periodic disturbance
Source: Sci Rep. 2015 Oct 20;5:15455. doi: 10.1038/srep15455 (PMC4612536; doi:10.1038/srep15455)
Supplement: Supplementary Information [file srep15455-s1.pdf]

Species extinction thresholds in the face of spatially correlated periodic disturbance

Jinbao Liao[a,*], Zhixia Ying[b], David E. Hiebeler[c], Yeqiao Wang[a], Takenori Takada[d], Ivan Nijs[e]

[a] Ministry of Education's Key Laboratory of Poyang Lake Wetland and Watershed Research, Jiangxi Normal University, Ziyang Road 99, 330022 Nanchang, China.

[b] State Key Laboratory of Vegetation and Environmental Change, Institute of Botany, Chinese Academy of Sciences, Beijing 100093, China.

[c] Department of Mathematics and Statistics, University of Maine, 333 Neville Hall, Orono, ME 04469, USA.

[d] Laboratory of Mathematical Ecology, Graduate School of Earth Environmental Science, Hokkaido University, 060-0810 Sapporo, Japan.

[e] Research Group Plant and Vegetation Ecology, Department of Biology, University of Antwerp (Campus Drie Eiken), Universiteitsplein 1, B-2610 Wilrijk, Belgium.

* Corresponding author: Dr. Jinbao Liao (jinbaoliao@163.com)

Tel.: +86-(0)791-88120537; Fax: +86-(0)791-88120538

Supplementary Information

Supplementary A – Derivation of local population dynamics

As $q_{I/I} = \rho_{II}/\rho_I$ (see Methods), we have the dynamics of local population density

$$\frac{dq_{I/I}}{dt} = -\frac{q_{I/I}}{\rho_I} \cdot \frac{d\rho_I}{dt} + \frac{1}{\rho_I} \cdot \frac{d\rho_{II}}{dt}, \tag{A.1}$$

in which the transition rate of doublet density ρ_{II} is

$$\frac{d\rho_{II}}{dt} = -2 \cdot \left(m + \frac{1}{z}\gamma + \frac{z-1}{z}\gamma \cdot q_{I/II} \right) \cdot \rho_{II} + 2 \cdot \rho_{IE} \cdot \left\{ \alpha \cdot \left[z^{-1} + \left(1 - z^{-1}\right) \cdot q_{I/EI} \right] + \beta \cdot \rho_I \right\}. \tag{A.2}$$

In equation (A.2), the first term denotes the transition of the pair *I-I*→*I-E* or *E-I* because of species mortality, including intrinsic mortality (*m*) and increased mortality by intraspecific competition. As a target individual *I* has four nearest neighbours (z=4) following Von Neumann neighbourship, species mortality caused by conspecific competition can be affected by the already presence of a neighbouring individual *I* in the pair of *I-I* sites (1/z), as well as the potential presence of conspecific individuals *I* from the remaining neighbours $(z-1)/z$. The second term describes the transition rate of the pair *I-E* (or *E-I*) becoming *I-I*, which can be achieved by both local and global dispersal. With local dispersal, species establishing the *E*-site in a pair of *I-E* can be realized by two possible ways: the local colonization from the individual *I* in the pair of *I-E*, and the potential presence of individuals from other z-1 neighbours of the target *E*. The parameter $q_{i/jk}$ (e.g., $q_{I/II}$ and $q_{I/EI}$; $i, j, k \in \{E, I\}$) represents the conditional probability for an *i*-site which is another neighbour of a *j*-site in the pair of *j-k*. According to the pair approximation[20] with $q_{i/jk} \approx q_{i/j}$, we can rewrite equation (A.2) as

$$\frac{d\rho_{II}}{dt} = -2\cdot\left(m + \frac{1}{z}\gamma + \frac{z-1}{z}\gamma\cdot q_{I/I}\right)\cdot\rho_{II} + 2\cdot\rho_{IE}\cdot\left\{\alpha\cdot\left[z^{-1} + \left(1 - z^{-1}\right)\cdot q_{I/E}\right] + \beta\cdot\rho_I\right\}, \qquad \text{(A.3)}$$

where

$$q_{I/E} = \frac{\rho_{IE}}{\rho_E} = \frac{\rho_{IE}}{1-\rho_I}\cdot\frac{\rho_I}{\rho_I} = \rho_I\cdot\frac{q_{E/I}}{1-\rho_I} = \rho_I\cdot\frac{1-q_{I/I}}{1-\rho_I}, \qquad \text{(A.4)}$$

with $q_{E/I} = \rho_{IE}/\rho_I$ and $q_{E/I} + q_{I/I} = 1$.

Combining equations (1), (A.1), (A.3) and (A.4), we can derive the dynamics of local population density $q_{I/I}$ as

$$\begin{aligned}\frac{dq_{I/I}}{dt} = {} & 2\cdot\left(1-q_{I/I}\right)\cdot\left\{\alpha\cdot\left[\frac{1}{z} + \frac{(z-1)\cdot\left(1-q_{I/I}\right)\cdot\rho_I}{z\cdot\left(1-\rho_I\right)}\right] + \beta\cdot\rho_I\right\} \\ & - q_{I/I}\cdot\left[\alpha\cdot\left(1-q_{I/I}\right) + \beta\cdot\left(1-\rho_I\right) + \left(m + \frac{2}{z}\gamma + \frac{z-2}{z}\gamma\cdot q_{I/I}\right)\right]\end{aligned}. \qquad \text{(A.5)}$$
